# Challenges in Whole Exome Sequencing: An Example from Hereditary Deafness

**DOI:** 10.1371/journal.pone.0032000

**Published:** 2012-02-21

**Authors:** Asli Sirmaci, Yvonne J. K. Edwards, Hatice Akay, Mustafa Tekin

**Affiliations:** 1 John P. Hussman Institute for Human Genomics, University of Miami Miller School of Medicine, Miami, Florida, United States of America; 2 Dr. John T. Macdonald Department of Human Genetics, University of Miami Miller School of Medicine, Miami, Florida, United States of America; 3 Radiodiagnostics Unit, Veni Vidi Hospital, Diyarbakir, Turkey; 4 Division of Pediatric Genetics, Ankara University School of Medicine, Ankara, Turkey; Stanford University School of Medicine, United States of America

## Abstract

Whole exome sequencing provides unprecedented opportunities to identify causative DNA variants in rare Mendelian disorders. Finding the responsible mutation via traditional methods in families with hearing loss is difficult due to a high degree of genetic heterogeneity. In this study we combined autozygosity mapping and whole exome sequencing in a family with 3 affected children having nonsyndromic hearing loss born to consanguineous parents. Two novel missense homozygous variants, c.508C>A (p.H170N) in *GIPC3* and c.1328C>T (p.T443M) in *ZNF57*, were identified in the same ∼6 Mb autozygous region on chromosome 19 in affected members of the family. Both variants co-segregated with the phenotype and were absent in 335 ethnicity-matched controls. Biallelic *GIPC3* mutations have recently been reported to cause autosomal recessive nonsyndromic sensorineural hearing loss. Thus we conclude that the hearing loss in the family described in this report is caused by a novel missense mutation in *GIPC3*. Identified variant in *GIPC3* had a low read depth, which was initially filtered out during the analysis leaving *ZNF57* as the only potential causative gene. This study highlights some of the challenges in the analyses of whole exome data in the bid to establish the true causative variant in Mendelian disease.

## Introduction

Hearing loss is one of the most common sensorial disorders in humans. Genetic factors account for more than 50% of cases with congenital or prelingual hearing loss, with autosomal recessive (77%), autosomal dominant (22%), and X-linked inheritance (1%) [1], [2]. Identification of the responsible mutation in families with autosomal recessive nonsyndromic hearing loss is difficult since there are mutations in 40 different genes identified for this common form of deafness (Hereditary Hearing Loss Homepage_http://hereditaryhearingloss.org/). A two step approach combining linkage analysis and whole exome sequencing based on the next generation technologies have been applied previously in other studies for Mendelian disease [3], [4] including deafness [5], [6]. The first step uses the genome wide SNP genotyping to identify autozygous regions when parental consanguinity is present and narrows down the search space for possible loci. The second step examines exome sequences to identify genetic variation at base-pair resolution and survey the protein coding portion of the human genome [7]. The integrated approach is faster and more cost efficient than the sequencing various candidate genes with the traditional Sanger sequencing techniques since the resulting loci generated from linkage are typically too large [3]. Thus whole exome sequencing using the next generation technologies provides a new and transformational approach for identifying causative mutations in Mendelian disorders [5], [7]–[12]. Here, we apply this two step approach, face the challenges, and eventually uncover a novel mutation causing hereditary hearing loss in a family. This study provides some comprehensive insights which would be valuable in certain scenarios and will help minimize certain limitations in using the new whole exome sequencing technologies.

## Materials and Methods

**Figure 1:**
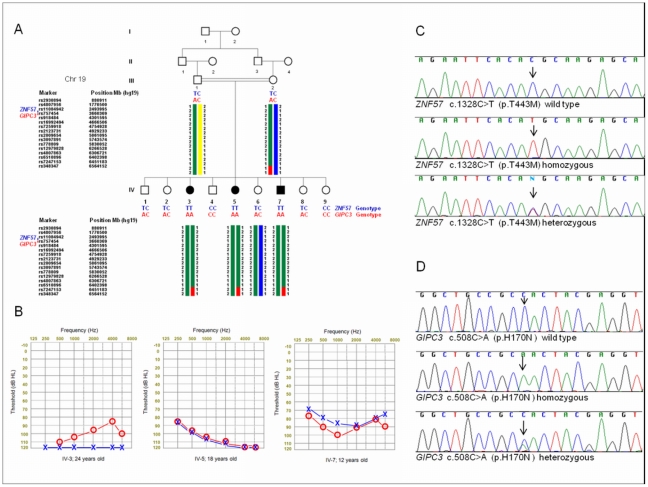
Pedigree with haplotypes, audiograms and two identified variants. (A) The pedigree and the third longest autozygous region on chromosome 19 that co-segregates with the phenotype. (B) Audiograms of affected members in the family. (C) Electropherograms showing the wild type, homozygous and heterozygous forms of the variant p.T443M in *ZNF57*. (D) Electropherograms showing the wild type, homozygous and heterozygous form of the variant p.H170N in *GIPC3*.

This study was approved by the Ankara University Medical School Ethics Committee (Turkey), and by the University of Miami Institutional Review Board (USA). All participants provided written informed consent prior to enrollment. Written informed consent was obtained from the next of kin on the behalf of the minors/children participants involved in this study. A family with three affected children who were diagnosed with sensorineural hearing loss via standard audiometry was recruited (Figures 1A and 1B). A thorough clinical evaluation including an ophthalmological exam and high resolution CT scans of the temporal bone in affected family members were normal. EKGs, liver and kidney function tests, serum electrolytes, urinalysis, CBC, and leukocyte subgroups were all within normal limits in affected subjects. DNA was extracted from peripheral leukocytes of each member of the family via a phenol chloroform method. Obtained samples were prescreened for mutations in *GJB2* (MIM 121011) via Sanger sequencing of both exons and for the m.1555A>G mutation in *MTRNR1* (MIM 561000). Heterozygous p.M163V amino acid change was found in *GJB2* in all three affected and three unaffected siblings and in father but no other *GJB2* mutations were present in the affected members.

Genome-wide SNP genotyping was performed in six members of the family (III-1, III-2, IV-3, IV-5, IV-6, and IV-7) using Affymetrix 6.0 arrays. Genotypes were transferred into Excel files and sorted according to genomic positions along with all 40 previously identified autosomal recessive nonsyndromic deafness genes. The co-segregation of the flanking genotypes for each gene was visually evaluated.

In order to identify the responsible variant, genomic DNA of IV-7 was evaluated by whole exome sequencing. The Agilent Human SureSelect 50 MB kit was used to extract the target regions from genomic libraries for exome sequencing. The sample was multiplexed with two other samples in a single lane of an Illumina HiSeq 2000 flow cell allowing for the sequencing of 101 bp paired-end reads. Raw data were analyzed using v1.7 of the Illumina CASAVA pipeline to extract the reads.

The reads were aligned with the human genome reference sequence (hg19 build), using the Mapping and Assembly with Quality (MAQ) software v0.7.1 [13]. Pairs of reads with identical outer coordinates were removed to improve the overall accuracy of variant calling. Variants (SNPs and indels) were called with MAQ. SNPs with a read coverage ≥8× and a Phred-like consensus quality of ≥20 were considered in the initial analysis. SNPs were annotated with SeattleSeq (http://gvs.gs.washington.edu/SeattleSeqAnnotation/) version 6.13 into functional categories such as missense, nonsense, splice sites, coding, non-coding, UTRs. DNA variants were filtered against dbSNP132 [14] and phase 3 of the 1000 Genomes project. PolyPhen2 predictions were generated for non-synonymous SNPs [15]. Indels with read coverage ≥8× covered by reads from both strands were predicted. Indels were annotated with SeattleSeq with respect to relative position of a gene (eg coding sequence, downstream or intronic) and their affects (eg frame-shift, amino-acid insertion or amino-acid deletion). After the initial analysis SNPs and indels were re-analyzed with read coverage ≥2× and ≥4× for comparison of the obtained variants.

**Figure 2:**
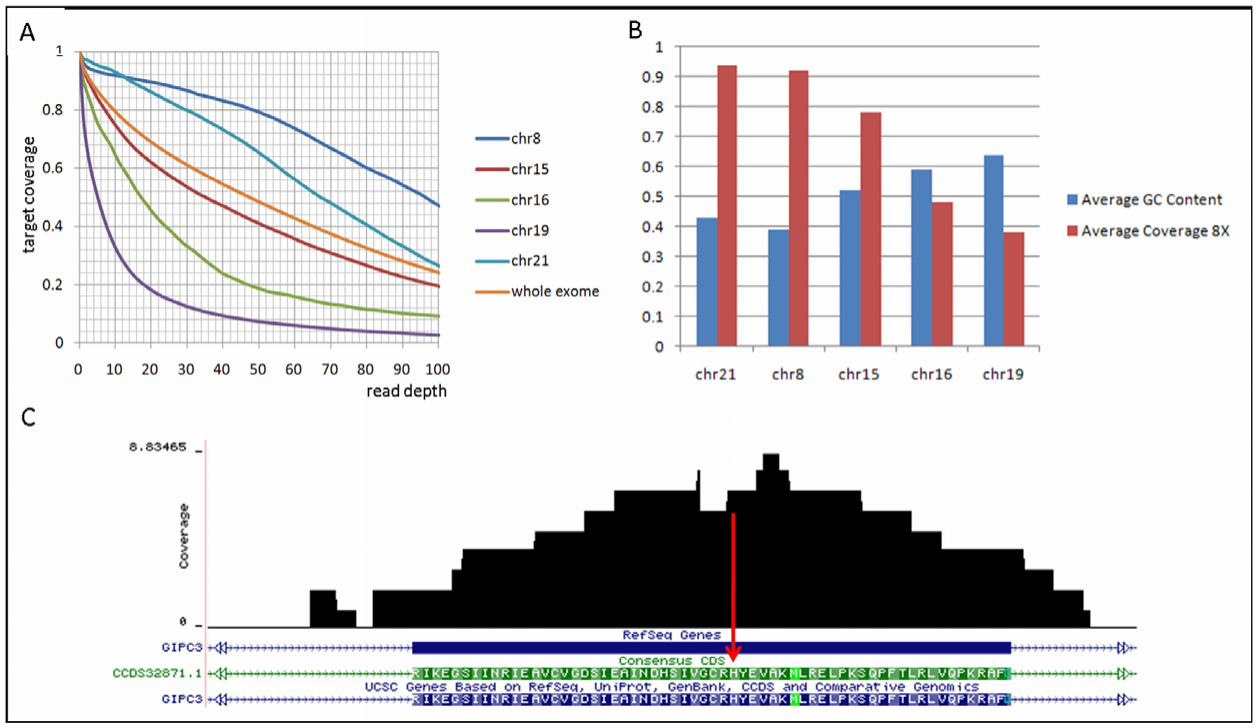
Coverage of autozygous regions with whole exome sequencing. (A) The exome coverage of the five longest autozygous regions. The plot shows the fraction of on-target coverage (Y-axis) and the read depth (X-axis) for the following specified regions. (B) Average coverage at minimum 8× and GC content of five autozygous regions. (C) Coverage of exon 3 in *GIPC3*. Red arrow indicates mutation point.

The Agilent Human SureSelect 50 MB whole exome capture and subsequent sequencing was evaluated by calculating the fraction of the target covered and the average read depth of the target. The MAQ alignments formed the basis of the depth and coverage calculations (Figures 2 A and 2B). On-target and off-target coverage were computed to create wig files that are uploaded into the UCSC Genome Browser [16] for visual assessment of depth and coverage and further annotate regions of interest (Figures 2C and S1).

Three-dimensional models were built using MODELER [17] from the Accelrys Discovery Studio package. BLAST sequence similarity searches [18] were used to identify suitable structural templates. This was done by using the query sequence and searching against the database of sequences from the Protein Data Bank (PDB) [19] using BLAST. The structural templates were selected by choosing the sequence of known structure with the highest percent identity with the query sequence. Software modules from the Accelrys Discovery Studio were used to inspect the molecules for quality control and to create illustrations.

## Results

Results of genome-wide SNP genotyping showed that none of the known deafness genes co-segregated with the phenotype. In the family, five autozygous segments longer than 2 Mb were present on chromosomes 8, 15, 16, 19 and 21 (Table 1). These five autozygous regions include 382 annotated genes.

**Table 1:** Five autozygous regions detected with Affymetrix 6.0 arrays in the family.

*Chromosome*	*Start Point (SNP)*	*End Point (SNP)*	*Autozygous Regions (hg19)with Affy 6.0 (6 family members)*	*Size (bp)*	Autozygous Regions (hg19) with exome sequencing in IV:7*	*Size (bp)*
15	rs16952059	rs12593367	68,718,000–81,335,100	12,617,101	74,364,703–81,410,488	7,045,785
8	rs2981099	rs7836491	73,855,527–80,351,787	6,496,261	71,040,655–86048011	15,007,356
19	rs8102615	rs7247153	1–6,451,433	6,451,433	105,101–4,511,278	4,406,177
21	rs11702247	rs2205081	38,699,159–41,708,994	3,009,835	38,568,308–45,651,413	7,083,105
16	rs9926500	rs4782341	85,880,671–88,649,755	2,739,084	85848265–89178474	3,330,209

To define autozygous regions from the exome sequences (*) the following filters were applied to reduce the incident of false positives (phred-like consensus score ≥100 and a minimum read depth of 20).

The exome sequencing experiment of one affected individual (IV-7) achieved the expected number of reads (87,586,240) and target coverage plus average read depth. Eighty seven million reads were generated which constitutes 8.6 gigabases of raw sequence. More than 95% of the reads mapped to the reference genome. Comparable with other labs [5], [9]–[11], [20], [21], when measured at a minimum depth of 8×, 82% of the target region was covered with an average depth of 68× (Figure 2A). Likewise, when measured at 1× and 20× coverage, nearly 95% and 69% of the intended target was covered with an average depth of 68× and 66× respectively. In terms of variant calls, the MAQ predicted 99,374 SNPs and 5,420 indels.

The five autozygous regions from genome wide genotyping data on chromosomes 8, 15, 16, 19 and 21 were investigated using the results of the exome sequencing. For the five autozygous regions much variation on the read depth was observed. Using a minimum depth of 8 as a filter, the chromosome 8 region has high average read depth of 101× with 92% coverage compared to the chromosome 19 region which has low average read depth of 15× with 38% coverage (Figure 2A). There was a strong negative correlation (r = −0.96) between the percentage of gunanine cytosine (GC) bases and the read depth or coverage of autozygous regions (Figure 2B). We focused on exonic and flanking intronic variants within these five autozygous regions. Table 2 shows the four novel homozygous missense, nonsense, splice site and frame shift variants (not reported in dbSNP132) in the five autozygous regions when we used a filter of minimum 8× read depth. Sanger sequencing confirmed one novel missense variant in the second longest autozygous region on chr8 (76476256A>T) in *HNF4G* (MIM 605966) and one novel missense variant in the third longest autozygous region on chr19 (2917947C>T) in *ZNF57* (no MIM number available) (nucelotide numbers are according to Hg19). We then recruited additional family members who were not typed with Affymetrix 6.0 chips to evaluate co-segregation of these variants. The novel variant c.1263A>T (p.Q421H) in *HNF4G* did not co-segregate with the phenotype in the entire family but variant c.1328C>T (p.T443M) in *ZNF57* did (Figure 1A and C). For the variant in *ZNF57* PolyPhen2 classification was possibly damaging with a score of 0.938, MutPred predicted that T443M amino acid substitution caused a gain of catalytic residue at V439 (p = 0.0472) and predicted the g score (probability of deleterious mutation) of 0.497 [15], [22]. Four coding exons and intron-exon boundaries of *ZNF57* were Sanger sequenced and no other nucleotide change was found. The indentified nucleotide change was not found in 335 Turkish controls via Sanger sequencing.

**Table 2:** Novel missense, nonsense, splice site, and frameshift variants in top five autozygous regions.

*Chromosome*	*Position (hg19)*	*Reference Base*	*Alleles*	*Accession Number*	*Variant Category*	*Amino Acids*	*Protein Position*	*Gene List*	*Phred Quality*	*Read Depth*	*Sanger*
8^a^	76,476,256	A	T/T	NM_004133	missense	GLN,HIS	421/446	*HNF4G*	255	118	+
15^a^	79,045,519	C	T/T	XM_929902	missense	ARG,CYS	43/86	*LOC646938*	75	16	−
15^a^	75,581,777	C	A/A	NM_001145224	missense	GLN,LYS	202/694	*GOLGA6D*	22	28	−
19^a^	2,917,947	C	T/T	NM_173480	missense	THR,MET	443/556	*ZNF57*	255	82	+
15	79,058,730	A	G/G	NM_014272	missense	SER,PRO	1175/1687	*ADAMTS7*	39	4	−
19	3,586,908	C	A/A	NM_133261	missense	HIS,ASN	170/313	*GIPC3*	42	5	+

a These variants were detected when filter for read depth was ≥8×.

ZNF57 is a recently discovered human zinc finger gene which has not been implicated in hearing loss. The ZNF57 protein product comprises 555 amino-acids with a KRAB-A domain at the amino-terminus and 13 tandemly arranged C2H2 zinc fingers at the carboxyl-terminus. Over expression of ZNF57 was shown to inhibit the transcriptional activities of NFAT and p21 demonstrating that ZNF57 is likely to function as a negative transcriptional regulator in NFAT-p21 signaling pathway [23]. The variant p.T443M is located in the linker between zinc fingers 10 and 11. The wild type linker in ZNF57 (has the sequence TQEQL) and the canonical zinc finger linker sequence is TGEKP. Both linkers comprise five residues and have a conserved threonine at the first position. Threonine at this position is highly conserved and attains a ConSeq conservation score of 8 in a scale of 1 to 9 (where 9 is most conserved) [24]. This conserved threonine is changed to methionine in the variant form of ZNF57 p.T443M. The functional unit for the zinc finger protein ZNF57 is unknown. Whilst zinc fingers are known to bind DNA, zinc fingers also interact directly with proteins [25] and RNA [26] and many have more than one role and form both protein-DNA and protein-protein interactions [25]–[29].

**Figure 3:**
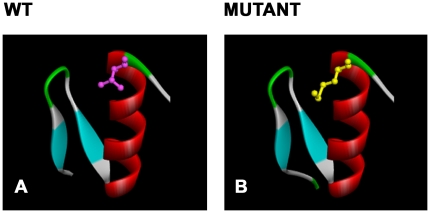
Molecular modeling of p.T443M in ZNF57. The zinc finger domain comprises two β-strands (blue) and one α-helix (red); the turns (green) and the loops (light gray) are shown. Amino-acid residue 443 is pink in the wild type (A) and yellow in the mutant (B).

The full-length ZNF57 sequence was BLASTed against the sequences of the PDB. A designed zinc finger peptide with six zinc fingers known as Aart [30] had the highest percent sequence identity with the query. The query sequence ZNF57 (with only the four zinc fingers 9, 10, 11 and 12) was aligned with the structural template Aart (PDB Code: 2I13 – chain B) using the Accelrys global alignment program. The alignment comprises 144 topologically equivalent position sharing 47.2% sequence identity (ZNF57:362–505 and 2I13:152–295). Indels were absent in the alignment. Protein structural models for ZNF57 (the wild type and p.T443M) were built (Figure 3). Threonine 443 of the wild type is the last residue at the C-terminal end of the α-helix and likely contributes to the α-helix cap (Figure 3). The methionine side chain is longer, more flexible and is unbranched compared with threonine (Figure 3). Mutation of threonine to methionine is likely to affect DNA binding capability indirectly in several different ways [31], [32]. Threonine is capable of being phosphorylated whereas methionine is not. Phosphorylation and dephosphorylation is implicated in the regulation of zinc finger protein binding and function and the conserved threonine in the linker region is a prime candidate for this type of regulation. Threonine is on the surface and accessible to possible phosphorylation events [31]. Others suggest that the DNA-induced helix capping in the conserved linker sequence is a determinant of binding affinity in C2H2 zinc fingers [32]. In evolution, threonine is one of the most frequently observed amino acids at this position in the zinc finger domain topology [26]. In mutational studies of the linkers between two contiguous zinc fingers, mutation of threonine to alanine had deleterious effects on DNA binding [33]. Similarly, mutating threonine to leucine in the linker was shown to reduce DNA binding [34].

While this work was ongoing a missense mutation in *Gipc3* was reported to be associated with age-related sensorineural hearing loss in the mouse, and two missense variants in *GIPC3* (MIM 608792) in two small families with sensorineural hearing loss [35]. The autozygous region on chromosome 19 in our family also includes *GIPC3*, in which no novel variant had passed our filters. We then re-analyzed the exome sequencing data reducing the filter for read depth to ≥4× instead of ≥8×; two additional variants in autozygous regions were detected (Table 2) and only the variant c.508C>A (p.H170N) in exon 3 of *GIPC3* was confirmed by Sanger sequencing (Figure 1D). Read depth for this variant was 5 and the exon containing this variant was poorly covered (Figure 2C). PolyPhen2 classification for this variant was probably damaging (score 1.0) and MutPred predicted the g score (probability of deleterious mutation) as 0.850. A ConSeq conservation score of 9 is obtained for H170 showing that this residue is highly conserved. The mutation was absent in 335 healthy ethnicity-matched controls.

**Figure 4:**
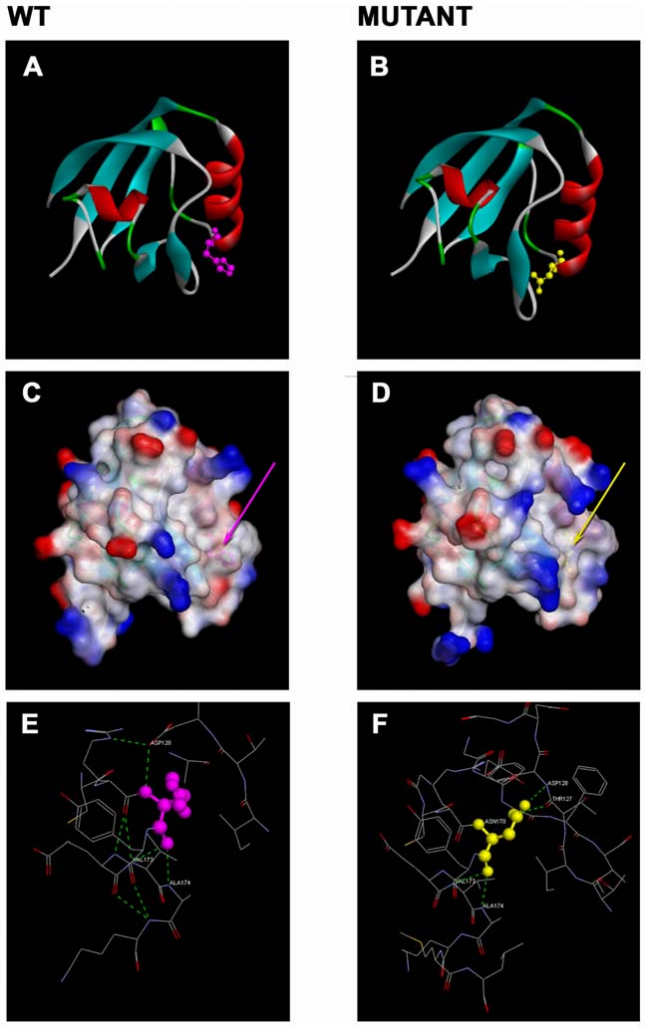
Diagrams of structural models for GIPC3. The ribbon diagrams for the 3D models of the PDZ domain in GIPC3. The wild type (A) and the mutated p.H170N (B) forms are shown. The key for structural features follow; the α-helices (red); the β-strands (blue); the β-turns (green); the loops (light gray); the side-chain for H170 in the wild type (pink) and the side-chain for N170 highlights the mutation (yellow). The surface diagrams show the surface topology and the interpolated charge distribution of the PDZ domain of GIPC3. Both the wild type (C) and the mutated p.H170N protein (D) are provided. The p.H170N mutated form shows one of the substrate molecular recognition pockets as being deeper with a larger volume compared to the wild type. The p.H170N mutated form shows the interpolated charge distribution as being reduced compared with the wild type. Structural and local environment for position 170 with the H-bond patterns are shown. The GIPC3 wild type (E) shows an absence of side-chain to main-chain H-bonds with the H170 side-chain; whilst the asparagine side-chain (yellow) in the mutated protein (F) forms side-chain to main-chain H-bonds.

The GIPC3 sequences (wild type and p.H170N) were each aligned with the PDZ domain in GIPC2 (PDB code: 3GGE - chain B). The alignment (GIPC3:108–199 and 3GGE:3–95) comprises 92 positions sharing 26.6% sequence identity with no indels. The structural template (3GGE) and the models each have two α-helices and six β-strands. Protein structural models for GIPC3 (the wild type and p.H170N) were built (Figures 4A and 4B). The mutated form of GIPC3 was compared with the wild type, structural differences were observed. In the mutated form of the model, the substrate molecular recognition pocket was larger and the associated charge distribution was reduced (Figures 4C and 4D) compared with the wild type. In the wild type H170 side chain is pointing away from the core and the resulting side chain is solvent accessible whilst the asparagine side chain 170 in the mutated form of GIPC3 points inwards towards the hydrophobic core and forms a tight network of H-bonds. The asparagine side chain forms two side chain H-bonds with two main chain atoms (ASP 128 NH: ASN 170 OD1 and ASN 170 OD2-HD22:THR 127 O) which renders the side chain solvent inaccessible. In addition, N170 forms two main chain to main chain H bonds (ALA 174: ASN 170 O and VAL 173 N:ASN 170 O). Residue 170 is the first residue of α-helix 2. In the wild type the side chain solvent accessibility for residue H170 is greater than 10%. H170 does not form side chain to side chain H-bonds within the PDZ domain (Figures 4E and 4F). Whilst the accepted amino acid residue substitution profile is variable at position 170 across the PDZ superfamily [36], [37], histidine for the GIPC family members at this position is invariant. This position 170 coincides with a key ligand binding pocket [36], [37] and the mutation from histidine to asparagine is predicted to alter the ligand binding site with a change of substrate specificity resulting in an adverse alteration in the protein function.

## Discussion

**Figure 5:**
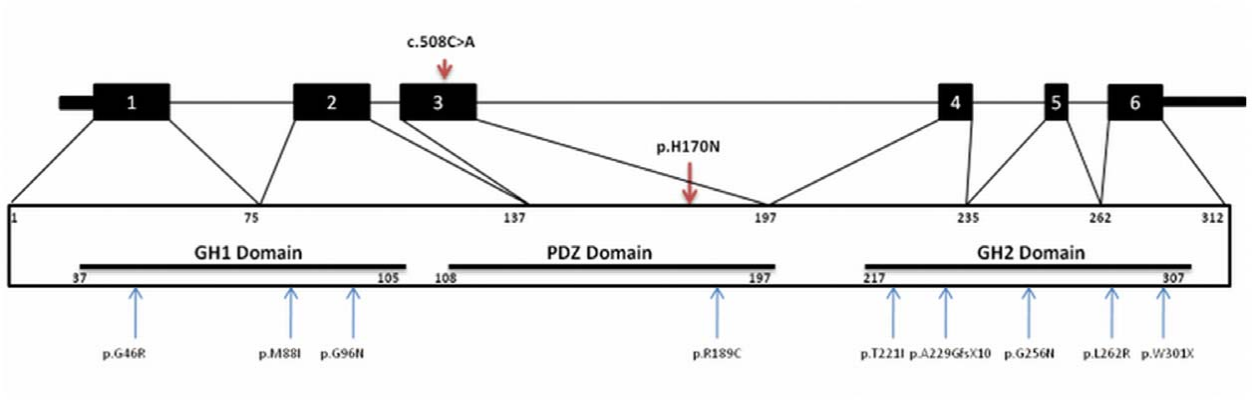
The two-dimensional structure of *GIPC3* and the localization of identified mutations.

After finding potential regions through autozygosity mapping we identified two novel rare variants to cause hearing loss in this study (GIPC3 p.H170N and ZNF57 p.T443M). Both variants were good candidates based on genetic and in silico data. It is important to restate that the first analysis was done using minimum read depth of 8× and the variant in *GIPC3* was not detected until a less stringent filtering was performed. If *GIPC3* was not known as a cause of deafness we could have concluded that the variant in *ZNF57* was causative. Human *GIPC3* gene encodes a 312 amino acid protein which localizes to the sensory hair cells in the inner ear [35]. In addition to the central PDZ domain, there are two conserved domains as GIPC homologous domain 1 (GH1 domain) and GH2 domain. The GH2 domain of GIPC1 interacts directly with the actin-based molecular motor myosin VI, in which mutations cause hearing loss in humans and mice [38]–[40]. After two missense mutations reported in one Indian and one Dutch family in *GIPC3* [35], very recently six more missense and a nonsense mutations were described in seven Pakistani families [41] (Figure 5). Following the previously reported p.R189C mutation in a Pakistani family, the p.H170N mutation in the Turkish family described in this study is the second mutation outside of one of the two GH domains. We should note that the mutation ZNF57 p.T443M might not be a red herring. We can speculate that this mutation also may contribute to hearing loss or more likely it might contribute to the susceptibility for another phenotype which was not considered here and that these two variants (ZNF57 p.T443M and GIPC3 p.H170N) are found to co-segregate. Whilst the *ZNF57* variant (NC_000019.9:2917947C>T, NM_173480.2:c.1328C>T, NP_775751.1:p.Thr443Met) is absent in dbSNP version 132, we note that in a recent update of the dbSNP (version 135), this variant is present as rs142727006 with an allele frequency of 0.003. The *GIPC3* variant (NC_000019.9:3586908) is absent from both the dbSNP (version 135) and the NHLBI Sequencing Project/Exome Variant Server Database. Our study clearly demonstrates some of the challenges faced using the high throughput exome sequencing technologies to find causative mutations in Mendelian disease. Table 3 shows the differences between analyzing the data using coverage filters of 2×, 4× and 8×. The ultimate goal of this combined approach is to identify disease causing mutations accurately and economically. To minimize the number of false negative results, an exome sequencing experiment requires adequate and uniform sequencing depth across the target regions. We demonstrate that despite attaining the expected quality metrics for the number of reads, the amount of DNA generated, the target coverage and the average depth across the intended target, a marked unevenness of capture of one region compared with other regions was evident (Figure 2). The targeted regions were not captured with uniformity (Figure S1) and we present this as an issue which needs to be evaluated with care when using exome sequencing as a tool for Mendelian disease gene discovery. Several factors can influence uniformity and unbiased capture and these include biases in the GC content as we clearly illustrate in this study (Figure 2). This study has provided some comprehensive insights and will be informative for scientists who plan to use exome sequencing technology. In certain scenarios this information may influence analysis or experimental design to reduce some of the limitations in surveying exome sequences for Mendelian disease gene discovery.

**Table 3:** Number of variants (SNPs) compared to the reference genome (hg19) with differing minimum depth filter and annotation categories.

*Filter Parameters*	*Whole Exome*			*Chr19:1-6,451,433*		
**Minimum depth filter**	2×	4×	8×	2×	4×	8×
**Total variants**	505,714	165,717	99,374	1,324	559	282
**Novel variants (Not reported in dbSNP132)**	244,726	61,182	24,594	730	287	119
**Novel missense, nonsense, splice site variants**	7,204	6,056	4,472	97	66	35
**Novel homozygous Variants**	3,644	149	108	5	2	1

## Supporting Information

Figure S1 **Coverage and read depth of five autozygous regions.**
(TIF)
