# Supplementary figures and images for: Challenges in Whole Exome Sequencing: An Example from Hereditary Deafness
Source: PLoS One. 2012 Feb 21;7(2):e32000. doi: 10.1371/journal.pone.0032000 (PMC3283682; doi:10.1371/journal.pone.0032000)

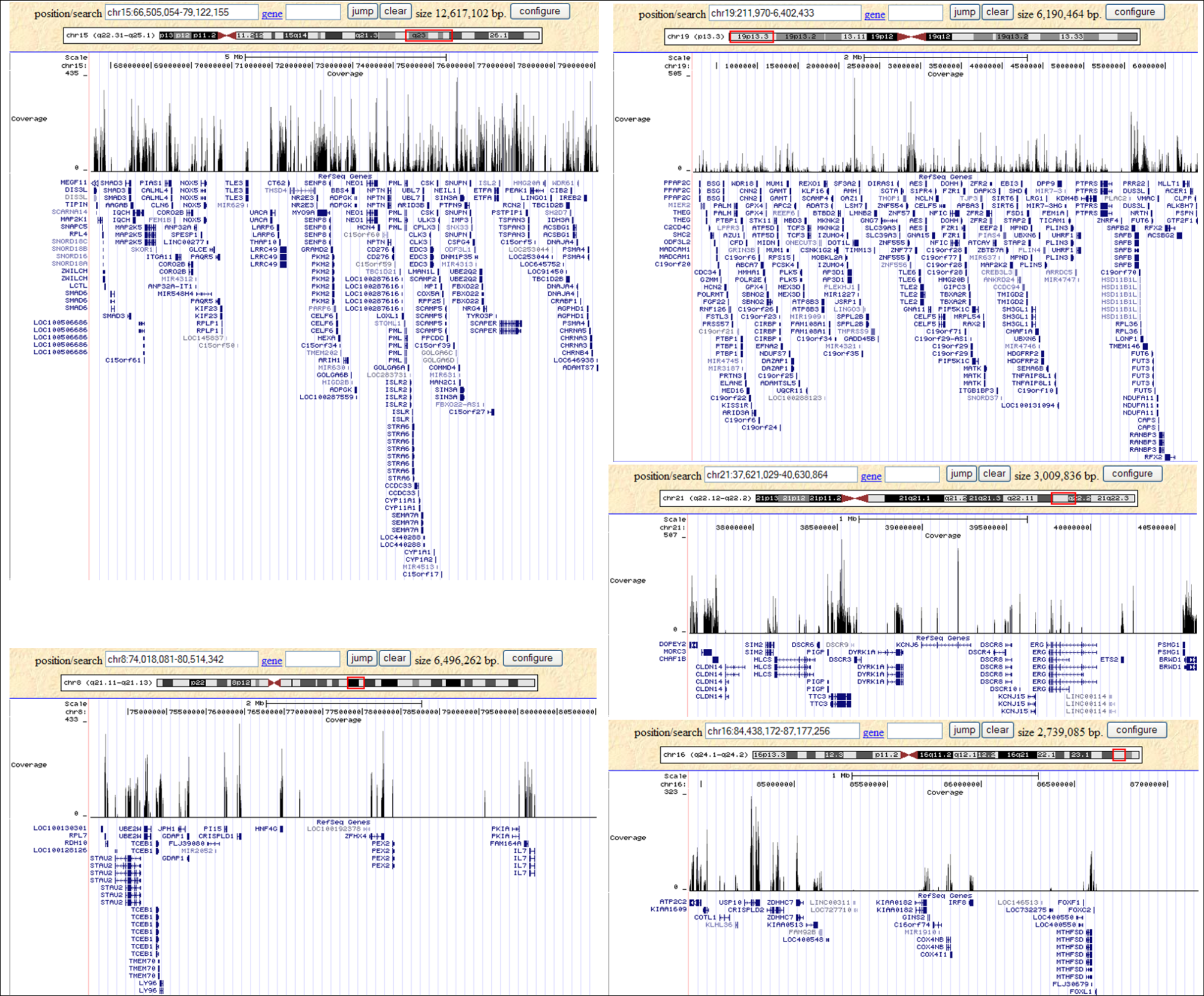

Supplement: Figure S1 — Coverage and read depth of five autozygous regions. (TIF) [file pone.0032000.s001.tif]
